# 

**Published:** 1865-04

**Authors:** 


### BOOKS AND PAMPHLETS RECEIVED

*Lectures on Surgical Pathology, delivered at the Royal College of Surgeons of England.* BY JAMES PAGET, F. R. S., *Surgeon Extraordinary to Her Majesty the Queen; Surgeon in Ordinary to His Royal Highness the Prince of Wales; Surgeon to St. Bartholomew's and Christ's Hospital. Revised and edited by* WILLIAM TURNER, M. D., *London,* F. R. C. S. E., F. R. S. E., *Senior Demonstrator of Anatomy in the University of Edinburgh. Third American edition. Philadelphia:* LINDSAY & BLAKISTON, 1865.

*The Pharmaceutist's and Druggist's Practical Receipt Book, with a Glossary of Medical Terms, and copious Index.* BY THOMAS F. BRANSTON. *Philadelphia:* LINDSAY & BLAKISTON, 1865.

*A Monograph on Glycerin and its uses.* BY HENRY HARTSHORNE, A. M., M. D.

*Alphabetical Index to Braithwaite's Retrospect, embracing Parts one to fifty*—1840—1865.

*A Radical Operation for Procidentia, read before the New York Obstetrical Society, December* 20, 1864. BY THOS. ADDIS EMMET, M. D., *Surgeon to the State Woman's Hospital, New York.*

*Twenty-Second Annual Report of the Managers of the State Lunatic Asylum, for the year* 1864. *Transmitted to the Legislature February* 4*th*, 1865.

### AMERICAN MEDICAL ASSOCIATION.

The Sixteenth Annual Session will be held in the city of Boston, on Tuesday, June 6th, 1865. The following Committees are expected to report:

On Insanity—Dr. H. K. Storer, Mass., Chairman.

On Exsection and its connection with Conservative Surgery—Dr. Lewis A. Sayre, New York, Chairman.

On Drainage and Sewerage of large cities and their Influence on Public Health—Dr. W. J. C. Duhamel, D. C., Chairman.

On Alcohol and its Relations to Man—Dr. G. E. Morgan, Md., Chairman.

On Quarantine—Dr. Wilson Jewett, Penn., Chairman.

On Medical Ethics—Dr. J. A. Murphy, Ohio, Chairman.

On the Microscope—Dr. James M. Corse, Penn., Chairman.

On the Relations which Electricity sustains to the Causes of Disease—Dr. Squire Littell, Penn., Chairman.

On the Morbid and Therapeutic Effects of Mental and Moral Influences—Dr. A. B. Palmer, Michigan, Chairman.

On the Causes of the Extinction of the Aboriginal Races of America—Dr. George Luckley, New York, Chairman.

On the Causes and Treatment of Ununited Fractures—Dr. Frank H. Hamilton, New York, Chairman.

On Diphtheria—Dr. Lucius Clark, Illinois, Chairman.

On the Uses and Abuses of Pessaries—Dr. James P. White, New York, Chairman.

On International Medical Ethics—Dr. J. Baxter Upham, Mass., Chairman.

On Climatology and Epidemic Diseases—Dr. C. W. Parsons, Rhode Island, Chairman.

On Autopsies in Relation to Medical Jurisprudence—Dr. T. C. Finnell, New York, Chairman.

On so-called Spotted Fever—Dr. James J. Levick, Pennsylvania, Chairman.

On the Introduction of Disease by Commerce and the Means for its Prevention—Dr. A. Nelson Bell, New York, Chairman.

On Patent Rights and Medical Men—Dr. David Prince, Illinois, Chairman.

On Prize Essays—Dr. D. H. Storer, Massachusetts, Chairman.

On Medical Education—Dr. T. Antiselle, D. C., Chairman.

On Medical Literature—Dr. Charles A. Lee, N. Y., Chairman.

On Necrology—Dr. C. C. Cox, Maryland, Chairman.

On Compulsory Vaccination—Dr. A. N. Bell, N. Y., Chairman.

On Ligature of the Subclavian Artery—Dr. Willard Parker, New York, Chairman.

On Revision of Plan of Organization—Dr. N. S. Davis, Illinois, Chairman.

On Specialists—Dr. J. Homberger, New York, Chairman.

On Medical Cups of Navy—Dr. T. L. Smith, N. Y., Chairman.

On Medical Cups of Army—Dr. C. S. Tripler, U. S. A., Chairman.

WM. B. ATKINSON, *Permanent Secretary.*

